# Cutaneous Dendritic Cells: Structure, Function and Immune Role

**DOI:** 10.3390/biomedicines14020460

**Published:** 2026-02-19

**Authors:** Ioana Cristina Alexandru, Mariana Grigore, Olga Simionescu

**Affiliations:** 1 1st Clinic of Dermatology Colentina Clinical Hospital, 020125 Bucharest, Romania; ioana.neo@gmail.com (I.C.A.); dana.simionescu@umfcd.ro (O.S.); 2 Department of Dermatology, “Carol Davila” University of Medicine and Pharmacy, 020021 Bucharest, Romania

**Keywords:** dendritic cells, antigen presenting cells, skin immunology, interleukins, T cells, Langerhans cells

## Abstract

Cutaneous antigen-presenting cells (APCs), particularly dendritic cells (DCs) and Langerhans cells (LCs), are a diverse population of cells that play a vital role in immune surveillance by initiating and shaping skin immune responses. They link innate and adaptive immunity by presenting antigens, migrating, and activating T lymphocytes, thereby acting as orchestrators of tissue immunity. This review provides an updated overview of the morphofunctional diversity of cutaneous APCs, ranging from epidermal LCs and DCs, to dermal conventional DCs (DC1/DC2), plasmacytoid DCs (pDCs), including newly defined subsets such as DC3, Axl^+^Siglec-6^+^ DCs (ASDCs) and LAMP3^+^ mature regulatory DCs (mRegDCs). Dynamic differences in APC composition and function between homeostatic and inflamed skin are discussed, with particular emphasis on inflammatory and autoimmune conditions such as psoriasis, lupus erythematosus and chronic atopic dermatitis, in which distinct DC subsets contribute to Th1 and Th17 immune circuits. This review is the first skin-related approach that extensively discusses the cutaneous role of APCs in the neuro-immune-cutaneous axis, as well as their interactions with the local microenvironment. Ongoing controversies regarding the classification and stability of certain DC populations are discussed. A better understanding of the diversity, migration mechanisms and microenvironmental interactions of cutaneous APCs could lead to the identification of new biomarkers and therapeutic targets for inflammatory, autoimmune, and oncological skin diseases.

## 1. Introduction

Antigen-presenting cells (APCs) represent an essential component of the immune system, being responsible for the capture and presentation of antigens to naïve T cells [1,2,3,4,5]. These include Langerhans cells (LCs), dendritic cells (DCs), dermal macrophages and B cells. All APCs present antigens, but only professional APCs can initiate adaptive immunity. The well-known distinction between professional and non-professional APCs is functional, not ontological.

Dendritic cells (DCs) were first identified as a distinct population in mouse lymphoid organs (Steinmann & Cohn, 1973) [1]. DCs serve as APCs, playing a central role in bridging innate and adaptive immunity [2,3]. They express high levels of MHC II molecules and are uniquely capable of priming naïve T cells, which makes them crucial for initiating adaptive immune responses [4].

In the skin, APCs form a specialised and heterogeneous network of resident cells distributed in both the epidermis and dermis. LCs are embryonic derivatives that originate initially from yolk sac-derived macrophage progenitors and subsequently from foetal hepatic progenitors. They constitute a distinct epidermal population with a unique developmental history and the ability to self-renew locally. LCs also play context-dependent immunoregulatory and immunostimulatory roles [5].

Under homeostatic conditions, Langerhans cells (LCs) constitute the main population of APCs in the epidermis.

However, recent analyses have revealed the presence of additional epithelial DC subsets in the steady-state epidermis [5,6,7,8,9]. By comparison, the dermis contains predominantly conventional DCs. In inflammatory settings, other populations are recruited or expanded, including plasmacytoid DCs, monocyte-derived inflammatory DCs, and inflammatory epidermal DCs [5,10,11].

Recently, single-cell and high-dimensional analyses have revealed the diversity of skin APCs, identifying additional populations that do not necessarily represent distinct subsets, but rather phenotypic and transcriptional variations. CD14^+^ dermal DCs are a heterogeneous population that includes both DC3 and monocyte-derived DCs [12,13,14]. Other emerging populations with distinct features have also been described, including Langerhans-like cells (migratory dermal cells), Axl^+^Siglec-6^+^ dendritic cells (ASDCs) and mature regulatory DCs (mregDCs), reflecting the expanding complexity of the skin dendritic cell compartment [12,15,16]. These emerging populations are of particular interest in skin pathology as they play a key role in chronic inflammation and tissue-specific pathogenesis in diseases such as psoriasis and atopic dermatitis [12]. In contrast, CD5^+^ DCs and conventional CD301b^+^ DCs do not constitute autonomous subsets, but rather reflect phenotypic variability within the dermal myeloid compartment [14,17,18].

Although their phenotypes are becoming clearer, important questions remain about how these subsets are maintained within the skin microenvironment, how they interact with keratinocytes, nerves, and the microbiome, and how they contribute to chronic inflammatory diseases.

## 2. Skin APCs and Migration

The maturation of DCs is a key process in activating the immune response. This process results from antigenic stimulation in the periphery [4,19,20,21].

**Figure 1:**
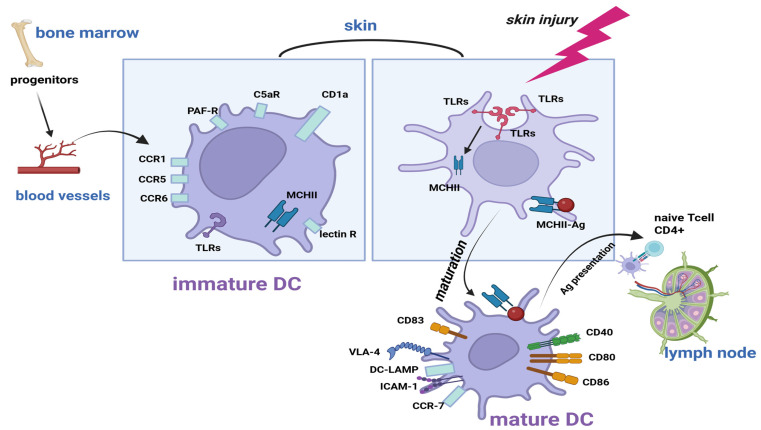
Migration and maturation of dendritic cells (DCs). Immature DCs originate from haematopoietic progenitors from the bone marrow [4,20] and are characterized by intense expression of CD1a, pro-inflammatory chemokine receptors (CCR1, CCR5 and CCR6), as well as other chemotactic receptors (PAF-R and C5a-R), intracellular MHC II molecules, Toll-like receptors (TLRs) and lectin-like receptors [19]. DC maturation occurs following the capture and processing of antigens (Ag) in the periphery [4,21]. Antigenic peptides-MHC II complexes are then transported to the cell surface to be recognized by and presented to T cells in the lymph nodes [23]. Mature DCs are characterized by increased expression of MHC II molecules on the cell surface [4,19,21], co-stimulatory molecules (CD40, CD80 and CD86) [4,19,21], maturation markers (CD83 and DC-LAMP) [19], adhesion molecules (VLA-4 and ICAM-1) [19,21,24] and the lymphoid chemokine receptor CCR7 [19,21,24]. Expression of pro-inflammatory chemokine receptors and phagocytic activity decreases [19,22]. Created in BioRender. Simionescu, D. [2026] https://BioRender.com/9j2vmtq.

Immature DCs originate from haematopoietic progenitors in the bone marrow [20]. These cells then circulate and migrate to peripheral tissues (e.g., skin, mucosal tissues and organs), where they play a role in the detection, capture and processing of antigens (Figure 1) [4,20]. Immature DCs exhibit high phagocytic activity and low secretion of pro-inflammatory cytokines [22].

The immunological mechanisms by which immature DCs take up antigens include phagocytosis (both opsonising and non-opsonising), pinocytosis, and receptor-mediated endocytosis [19,24,25].

Mature DCs migrate from tissues to lymph nodes and are professional APCs with a proinflammatory phenotype [4,20,21,22]. They migrate to secondary lymphoid organs via a complex interaction involving pro-inflammatory cytokines (TNFα, IL1β, IL16 and IL18) [19,26], chemokines and their receptors [19,21,24,27,28,29,30] (Table 1), prostaglandins [27], leukotrienes [19], intercellular adhesion molecules [19,21,27,31,32,33] and extracellular matrix proteins [28,34].

The lymphatic endothelium expresses the adhesion molecule CD112, which has recently been shown to play a role in the migration of mature dermal DCs [33]. Decreased expression of the proinflammatory chemokine receptor CCR6 allows mature DCs to leave the skin tissue, whereas increased expression of the lymphoid chemokine receptor CCR7 directs them to the lymph nodes [21,24]. LCs comprise distinct steady-state and activated subsets, a heterogeneity that underlies their variable maturation and migratory behaviour [8].

## 3. APC-Mediated T Cell Activation

**Figure 2:**
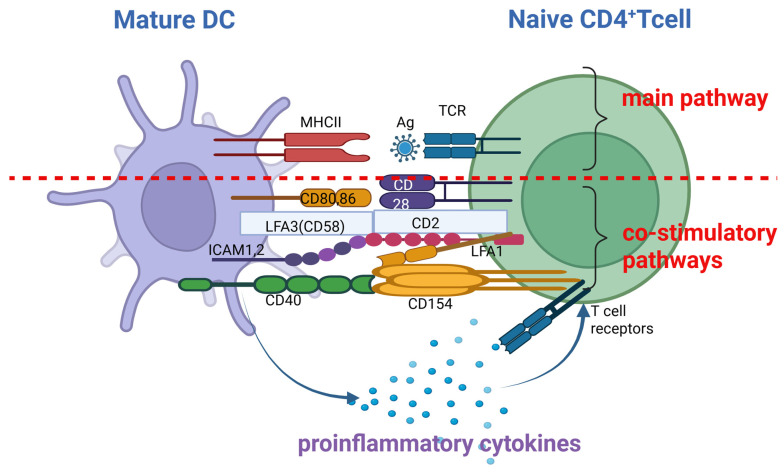
Activation of naïve CD4^+^ lymphocytes by mature DCs in lymph nodes. Interaction between mature DCs and naïve CD4^+^ Th cells occurs through three activating signals: a. Signal I, or the main signal, involves antigen-specific binding between the TCR and the antigen-MHC II complex on the DC surface; b. Signal II involves co-stimulatory interactions formed by the following pairs of molecules between the T cell and the DC—CD2:LFA-3, LFA-1:ICAM-1/ICAM-2, CD154:CD40, CD28:CD86/CD80; c. Signal III is represented by secretion of soluble mediators (pro-inflammatory cytokines) [19,23,35,36,37]. Created in BioRender. Simionescu, D. [2026] https://BioRender.com/k3fz3mo.

In lymph nodes, mature DCs and activated LCs activate naïve CD4^+^ T helper (Th) cells in three stages: antigen presentation, antigen recognition by T cells and formation of three activating signals (Figure 2).

The first signal involves the antigen-specific binding between the T-cell receptor (TCR) and exogenous antigen-MHC II complexes [19,23,35,36]. The second signal is exerted by co-stimulatory molecules expressed by mature DCs and activated LCs, which are recognized by corresponding molecules on Th cells, forming the following pairs: CD80/CD86:CD28, LFA3:CD2, ICAM1/ICAM2:LFA1 and CD40:CD154 [19,23,35,36,37]. For molecules without a peptide structure, such as lipids, the interaction between APCs and T cells is mediated by CD1 [19,23,38]. The third signal is provided by soluble mediators (proinflammatory cytokines) [19,23,35,36]. Apart from the type of APC, the dynamic profile of the signal delivered to TCR and costimulatory receptors is also important in order to differentiate Th cells [23,25,36].

Alongside these mechanisms, particular attention is given to cross-presentation: an essential immune phenomenon whereby DCs activate CD8^+^ T cells by presenting foreign antigens via MHC class I molecules [19,23,25,35]. Furthermore, cross-presentation is involved in the development of self-perpetuating lesions that are characteristic of chronic plaque psoriasis. While classical MHC class I molecules present endogenous antigens to CD8^+^ T cells, cross-presentation involves exogenous antigens, such as streptococcal proteins [19].

## 4. Skin APCs and Pattern Recognition Receptors (PRRs)

In dermatology, two families of PRRs are relevant to the biology and functions of skin APCs: C-type lectin receptors (CLRs) and Toll-like receptors (TLRs) [39,40].

CLRs are the defining PRRs for skin APCs as they enable recognition of both pathogen-associated structures and endogenous ligands released in response to stress or tissue injury [41,42,43]. LCs express the langerin/CD207 receptor, a specialised CLR that is involved in antigen internalisation and the organisation of characteristic Birbeck structures. This facilitates antigen processing and presentation in the epidermis and plays a role in developing immunity against pathogenic microorganisms [41,42]. Langerin/CD207 plays an essential role in protecting against HIV-1 infection by capturing and degrading virions, thus preventing T cell infection [42,43]. Dectin-1 (CLEC7A) is expressed by both LCs and dermal DCs and is involved in recognising bacterial and fungal components. It plays a role in generating specific immune responses to control these infections [41,42,43]. By recognising commensal/pathogenic bacterial antigens, fungal structures and endogenous alarmins, CLRs expressed by LCs and dermal DCs activate immune pathways that initiate pathogenic changes in psoriasis [41,42]. In atopic dermatitis, DC-SIGN (CD209) expression increases in DCs, promoting disease exacerbation by capturing allergens and inducing Th2/Th22 polarization. Conversely, DC-SIGN interaction with certain probiotics can promote regulatory T cell (Treg) responses and may have a protective effect [41]. These receptors are also involved in tumour antigen recognition, which is important in melanoma. Here, dectin-1 (CLEC7A) and DC-SIGN (CD209) activate cytotoxic antitumour responses dependent on dermal DCs [42,43]. Overall, CLRs define the specialised functions of skin APCs, complementing TLR-mediated responses and contributing to the modulation of skin immune responses [41,42].

**Figure 3:**
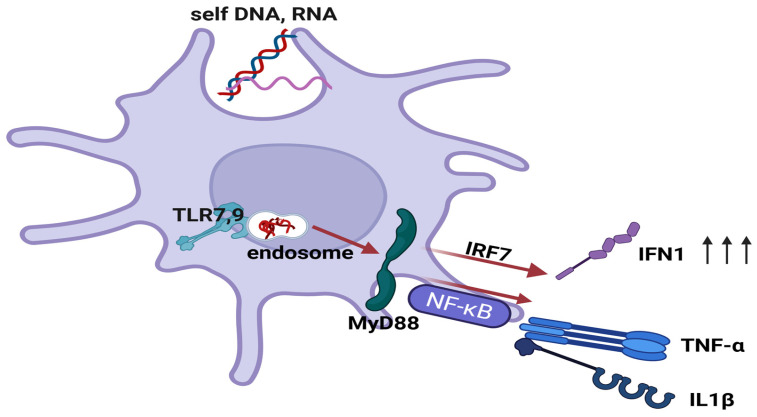
Activation of TLRs 7 and 9 by endogenous ligands (autoantigens). TLRs 7 and 9 can be activated by self-DNA and RNA fragments, causing the activation of the nuclear transcription factors NF-κB and interferon regulatory factor 7 (IRF7) via the MyD88 gene. IRF7 activation leads to increased synthesis of type I IFN, while NF-κB activation induces transcription of several pro-inflammatory cytokines and antimicrobial peptides. This aberrant activation of TLRs by self-derived molecules contributes to the initiation of inflammatory autoimmune disorders such as psoriasis or lupus [44,45,46,47,48]. Created in BioRender. Simionescu, D. [2026] https://BioRender.com/gr4lfk1.

Although expressed by numerous immune and non-immune cells, TLRs remain important sensors for human APCs. Humans have ten TLRs, which are either transmembrane (TLRs 1, 2, 4, 5, 6 and 10) or intracellular (TLRs 3, 7, 8 and 9). They use these receptors to detect both PAMPs and DAMPs, which activate the innate immune system [39,44]. The engagement of TLRs triggers the activation of the nuclear transcription factors NF-κB and IRF7 via MyD88. This process promotes the transcription of proinflammatory cytokines, antimicrobial proteins, and type I IFN (mainly IFN-α and IFN-β) synthesis [44,45,46]. This mechanism can be triggered by both exogenous and endogenous ligands, the latter of which activate endosomal TLRs. This phenomenon has important implications for inflammatory skin pathology (Figure 3) [40,45,47,48].

TLR stimulation also activates components of the AP-1 protein family via the TRAF-6/TAK-1/JNK-p38 signalling pathway [49]. AP-1 proteins, such as c-Jun and JunB, modulate the differentiation of immune cells and the production of inflammatory cytokines in response to TLR stimulation [49,50]. Psoriasis-like skin changes have been reported in mice with concomitant deletion of the c-Jun and JunB proteins in the epidermis [49]. Novoszel et al. propose that the expression of CCL2 and IL23 in activated DCs is immunologically controlled via activation of the TLR7/c-Jun/AP-1 axis. CCL2 is essential for the migration of plasmacytoid DCs to the lesion site, and c-Jun/AP-1 regulates its synthesis in DCs. Interestingly, c-Jun/AP-1 deletion in DCs reduces imiquimod-induced skin inflammation, suggesting that this pathway could be a therapeutic target for TLR-mediated dermatoses [49].

## 5. The Main Populations of Skin APCs

The skin APC population is heterogeneous. It comprises resident cells, such as LCs and conventional dermal DCs, as well as additional populations that are recruited during inflammation, such as plasmacytoid DCs and inflammatory epidermal and dermal DCs. While these are the main populations, the diversity of cutaneous APCs is greater, with new subsets still being described [5,10,11].

### 5.1. Resident APC Populations

#### 5.1.1. Langerhans Cells

LCs were first described by the medical student Paul Langerhans in his work Über die Nerven der menschlichen Haut (On the Nerves of the Human Skin, 1868) [51]. Due to their dendritic appearance, Langerhans initially equated them with nerve receptors. However, it is now clear that LCs are specialised APCs located in the basal and suprabasal layers of the epidermis and at the level of the follicular infundibulum [10,11,52,53].

Although LCs were traditionally considered to be the only APC in the epidermis [5,54], they are now recognized as a distinct and well-defined epidermal immune cell population. LCs account for 2–4% of the epidermal cell population, with a density of 500–1000 cells/mm^2^ [52,55]. LCs are stellate in shape with thin, long extensions. Their cytoplasm contains both common organelles and tennis racket-shaped Birbeck granules, which give them their characteristic appearance under an electron microscope [10,11,52,54]. Ontogenetically, LCs are of myeloid origin [52,56,57]. LC precursor cells originate from haematopoietic precursor cells in the yolk sac (macrophages) and the foetal liver (monocytes), which infiltrate the epidermis during late embryogenesis [54,58,59]. LCs are stable cells with a long lifespan and the ability to self-renew independently of CD34^+^ myeloid precursors [52,53,60,61]. Although LCs undergo ontogenesis that is similar to that of tissue macrophages and are currently recognised as a distinct subset of the mononuclear phagocyte system, they retain functions that are characteristic of DCs, especially under inflammatory stress [9,62,63]. The network of LCs develops and is maintained via cytokines and growth factors, such as TGFβ1 [5,54,62,64,65], IL-34 [5,54,62,66,67,68], CSF-1 [5,54,62,66], BMP-7 [5,69] and AXL [5,70], produced by epidermal keratinocytes. LCs form a complex network with keratinocytes via E-cadherin and other adhesion molecules [52,53,71].

#### 5.1.2. Epidermal Non-Langerhans DCs

In addition to LCs, recent studies have demonstrated the presence of other DCs in the epidermis under homeostatic conditions. These cells are distinct from classical LCs, both phenotypically and functionally. They express CD11c**, but lack Birbeck granules. They can also be distinguished from DCs by their epithelial localisation and higher CD1a expression, relative to dermal DCs [6,7,8,9]. Under inflammatory conditions, the epidermal DC compartment expands and diversifies to give rise to specialised inflammatory populations, including inflammatory epidermal dendritic cells (IDECs) [10,11].

LCs are classically characterised by their intense expression of the CD1a* and CD207/langerin** markers [10,11,52,72]. In order to differentiate LCs from other epidermal DC subsets, the analysis of additional markers is required, such as CD11b, CD11c and CD206. Human LCs typically exhibit low levels of CD11c/CD11b and lack CD206 [6,7,8,73,74].

#### 5.1.3. Conventional Dermal DCs (cDCs)

A second population of APCs found in a physiological context are dermal cDCs. These are mobile cells with a dendritic appearance [10,11,75]. They are distributed perivascularly in the superficial dermis [10,11]. Ontogenetically, they are also of myeloid origin, being derived from CD34^+^ haematopoietic stem cells. These cells then form CDPs. These then differentiate into pre-DCs at the medullary level—cells that leave the bone marrow and enter the circulation. They settle in lymphoid and non-lymphoid tissues, completing their differentiation process [24,53,56,76]. The maintenance of these cells in the dermis is sustained by signalling via the FLT3L-FLT3 and GM-CSF (CSF2)-CSF2R (CD116) axes [5,56,77,78].

The characterisation of cDCs relies on identifying specific surface markers. As the CD11c marker is conserved across species and identifies all myeloid-derived DCs as well as other APCs, such as macrophages, more specific markers have been tested to identify human DC subsets. Based on these markers, cDCs are categorised as either cDC1 or cDC2 [79].

In physiological conditions, conventional type 1 dermal DCs (cDC1s) represent only 10% of dermal DCs [5,10,79]. One of the initial markers used to identify cDC1, CD141 (BDCA3)*, has limitations because it is expressed by both cDC2 and monocytes, both in tissues and in culture. Currently, the markers considered most reliable for identifying human cDC1 are CLEC9A, CADM1, and XCR1 [14,74,80,81].

Conventional type 2 dermal DCs (cDC2s) represent the main dermal DC population (90%) and are identified by their preferential expression of CD11c** and CD1c (BDCA1)* [5,10,74,82]. cDC2 can also express low levels of CD207/langerin** [9,13,53,74].

Beyond their classical role in antigen presentation, in murine models, LCs can exchange antigens and even acquire MHC II molecules from dermal DCs in vivo under steady-state conditions [83]. This bidirectional exchange increases the range of antigens that LCs can present to T cells, thereby amplifying or modulating local immune responses. While LCs can promote immunity, they can also induce Tregs and contribute to tolerance. This suggests that the transfer of antigens between LCs and dermal DCs may influence the balance between activation and regulation. Disruption to this process has been linked to imbalances in skin immune homeostasis and may lead to chronic inflammatory skin diseases [83,84]. Understanding this antigen-sharing mechanism could lead to the development of therapeutic strategies aimed at restoring immune balance in disorders such as psoriasis and atopic dermatitis.

### 5.2. APC Populations in Skin Pathology (Inflammatory Context)

#### 5.2.1. Langerhans Cells in Skin Inflammation

**Figure 4:**
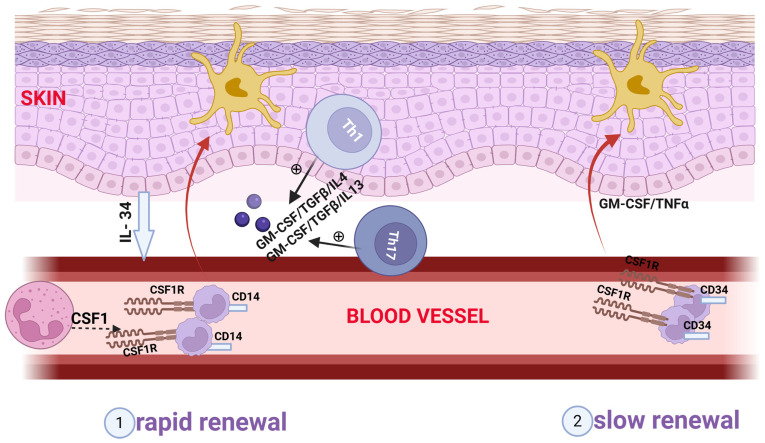
The two mechanisms of epidermal repopulation with Langerhans cells in conditions of severe tissue injury: a. A rapid, but transient mechanism, transforming circulating monocyte precursors/CD14^+^ monocytes into cells with short lifespan in the presence of GM-CSF/TGFβ/IL4 or GM-CSF/TGFβ/IL13 b. A slower, but stable mechanism generates a cell population with long lifespan from BM-derived CD34^+^ precursors in the presence of GM-CSF/TNFα [10,11,54,57,72,85]. Post-injury repopulation depends on CSF1R, which is expressed by the precursors, and its neutrophil-derived ligand CSF1, which promotes the migration of circulating precursor cells/monocytes to the epidermis. The maintenance of the LC population is also supported by keratinocyte-derived IL34 [66,71]. Red arrows-the transformation of precursors in LC; black arrows-cytokine release from TH1/Th17; dotted arrow-CSF1 release from neutrophils; blue arrow-Il-34 release from keratinocytes; +-stimulating effect. CSF1: colony-stimulating factor 1; CSF1R: colony-stimulating factor 1 receptor; CM-CSF: granulocyte-macrophage colony-stimulating factor. Created in BioRender. Simionescu, D. [2026] https://BioRender.com/00yk64q.

The first difference between inflamed and normal skin is that LCs migrate from the former to secondary lymphoid organs, where they present antigens. This migration process results in a reduced number of LCs and an altered distribution pattern within the epidermis [54,62,72,75]. The maintenance of the epidermal LC population relies on local proliferation under steady-state conditions or following minor trauma, or on the recruitment of circulating CCR2^+^ myeloid precursors following severe inflammatory injuries [52,54,61,62,71]. Consequently, the local capacity for self-renewal is lost, but repopulation occurs through two main mechanisms (Figure 4).

The first mechanism is rapid and transient, converting circulating monocyte precursors/CD14^+^ monocytes into a transient population of cells. The second mechanism is slower but generates a stable cell population resulting from bone marrow-derived CD34^+^ precursors [10,11,54,57,72,85].

**Figure 5:**
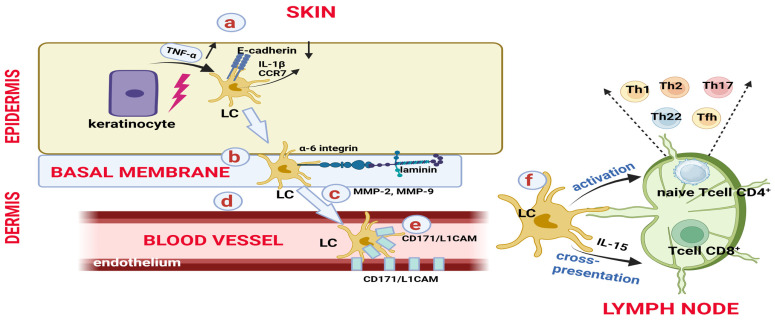
Migration of CCR7^+^ Langerhans cells from the epidermis to the lymph nodes. The migration of LCs to the lymph nodes is a multi-step process [62]: a. detachment of LCs from the network formed with neighbouring keratinocytes through downregulation of E-cadherin expression and the release of TNFα (from keratinocytes) and IL1β [28,52,62,75]; b. crossing of the basement membrane via binding of integrin-α6 to laminin [28,86]; c. guidance toward the dermis through the basement membrane, supported by the release of MMP-2 and MMP-9 (from LCs), which degrade collagen IV [28,52], as well as by fibroblast-derived CXCL12, which interacts with CXCR4^+^ LCs [52,54,62,87]; d. passage through the dermis; e. transendothelial migration via L1 adhesion molecules (CD171/L1CAM) [28,88]; f. CCL19/CCL21–CCR7 [28,29,52,54,62] and CXCL12–CXCR4 [28,31,54,71] pairs direct LCs from the dermis to lymphatic vessels and subsequently to the draining lymph nodes. In the lymph nodes, LCs preferentially induce the proliferation of CD4^+^ T cells, which then differentiate to secrete Th1 [89,90], Th2 [53,89,91,92,93], Th17 (via IL-15 and IL-6) [5,71,94,95], Th22 (via CD1a) [53,96] or Tfh cytokines [5,97,98]. Created in BioRender. Simionescu, D. [2026] https://BioRender.com/pvpna0c.

Although epidermal repopulation with LCs occurs through two main mechanisms, the migration of LCs from the skin to the lymph nodes is a multi-step process [62], controlled by IL-1β (derived from LCs and keratinocytes) [11,52,54,62,71], IL-18 [54,62,71] and TNF-α (derived from keratinocytes) [11,52,54,62,71,75] (Figure 5).

LCs are also involved in the cross-presentation phenomenon and can promote cytotoxic T cell responses via IL-15 release. Additionally, RANK (LC)-RANKL (keratinocyte) signalling induces the proliferation Tregs in the skin, thereby contributing to immune tolerance [75,99]. Conversely, LCs activated by exogenous pathogens cause the activation and proliferation of skin-resident memory T cells [99]. LCs also contribute to the early reactivation of resident memory CD8^+^ T cells in the epidermis [100]. In vitro, human LCs exposed to Candida albicans antigens have been shown to induce a greater number of effector T cells than Treg cells [79,99]. This suggests that the type of immune response induced by LCs depends on external signals [101]. Recent data from single-cell analyses have demonstrated that human LCs do not form a uniform compartment, but rather comprise several distinct subsets (steady-state LC1 and LC2, activated LC and migratory LC) with different transcriptional programmes, activation levels and immune responses. This highlights their functional plasticity and variable contribution to skin inflammation [8,63].

LCs secrete neurotrophins and neurotrophic factors, such as NGF and GDNF, which modulate skin neuronal activity. Decreased secretion of these molecules by LCs leads to a decreased density of peripheral nerve fibres in the epidermis and dermis—a finding that is classically observed in neuropathies. There are bidirectional links between the peripheral nervous system and LCs; for instance, locally released neuropeptides reduce the ability of LCs to present antigens in lymph nodes [102].

#### 5.2.2. The Pathology of Conventional Dermal DCs

cDC1s induce the proliferation of effector T cells and produce pro-inflammatory cytokines [5,24]. Upon activation of cellular immunity, they present exogenous antigens and secrete IL-12. By presenting these antigens, cDC1s activate CD4^+^ Th1 cells [24,74]. cDC1s are also involved in cross-presenting exogenous antigens to CD8^+^ T cells. They play a role in antiviral and antitumour immune defences, as well as in the development of post-vaccine immunity [5,24,74,80,103].

cDC2 plays a role in immune surveillance at the dermal level by presenting antigens to naïve CD4^+^ T cells [5,24,74]. Maturation-inducing stimuli (IL-1β, IL-6, TNF and PGE2) enhance these cells’ ability to induce T cell proliferation [10,79,82].

In inflammatory conditions, a CD11c^+^CD1c^+^CD5^+^CD14^−^ subset of skin DCs displays enhanced pro-inflammatory capacity, producing IL-12 and IL-23 and accumulating in psoriatic lesions. Single-cell analyses showed that CD5 does not define a distinct DC subset but reflects variability along the cDC2 compartment (being highly expressed on cDC2 and low/absent on DC3) and also marks a fraction of LCs [14,17]. Thus, CD5^+^ DCs represent a pro-inflammatory subset within cDC2 (and partly within LCs) and are associated with Th1/Th22 responses in inflammatory dermatoses.

CD301b^+^CD11b^+^CD11c^+^ DCs exhibit a strong pro-inflammatory profile and can drive an IL17-dependent psoriasis-like response. They represent an activated cDC2 subpopulation in inflammation rather than a distinct DC subset [18].

The activity of cDC2s is also influenced by the peripheral nervous system. Nerve endings come into contact with cDC2s, thereby modulating their ability to generate Th17-type immune responses and IL-23 synthesis via calcitonin gene-related peptide (CGRP) [104,105].

#### 5.2.3. Type 3 DCs (DC3)

In inflammation, DC3 (Table 2) represents a distinct, transcriptionally defined human DC subset characterised by CD1c^+^CD14^+^CD11c^+^CD163^+^CD5^−^ expression, which clearly separates them from cDC2 [12,14,53]. Unlike the heterogeneous CD14^+^ DC pool that includes both DC3 and monocyte-derived DCs, DC3 maintain a stable transcriptomic identity [13,14]. DC3 are found in circulation and in the dermis under steady-state and inflammatory conditions [14,53].

The contrast between cDC2 and dermal CD14^+^ DC3 highlights the functional diversity of skin DCs. cDC2 efficiently prime naïve CD4^+^ T cells, whereas CD14^+^ DC3 are less effective at T-cell priming but promote B-cell activation and regulatory T-cell induction [90]. Single-cell analyses in psoriasis further showed IL1B and IL23A co-expression in DC3 but not cDC2, emphasising their distinct contribution to inflammation [12]. Together, these subsets act complementarily, with cDC2 shaping adaptive T-cell responses and DC3 supporting chronic inflammation and humoral/regulatory pathways [12,53,79,90,106]. Deuterium-labelling studies also indicate kinetic similarities between circulating cDC2 and DC3, although it remains unclear how this behaviour translates within tissues such as the skin [16]. Recent studies indicate that DC3s may be involved in the pathogenesis of hidradenitis suppurativa (HS) by co-producing high levels of IL-1β and IL-23, acting as a crucial link between innate and adaptive immunity [12,107,108]. The IL-1β produced by these cells drives local inflammation, while IL-23 stimulates Th17 cells to produce IL-17. This results in the characteristic cytokine signature (TNF-α, IL-1β, IL-23 and IL-17) found in HS lesions.

#### 5.2.4. Inflammatory Epidermal DCs (IDECs)

IDECs are observed in humans in pathological contexts, and are present in chronic skin diseases, including atopic dermatitis, psoriasis, allergic contact dermatitis, mycosis fungoides and lichen planus. IDECs have a myeloid origin and differ from LCs in terms of their electron microscopic appearance (IDECs lack Birbeck granules) and their surface molecule profile (IDECs are CD206^+^, whereas LCs are CD206^−^) [73,109,110]. The fundamental immunological function of IDECs is to act as adaptive inflammatory APCs, essentially connecting innate and adaptive immunity. These cells emerge de novo in the inflamed epidermis and modulate T cell polarisation depending on the tissue context [10,11,73,110].

From an immunological point of view, IDECs play a role in IgE-dependent antigen presentation to T lymphocytes in atopic dermatitis, thus playing a central role in maintaining and amplifying chronic inflammation [73,110]. In psoriasis, IDECs activate the IL-23/Th17 inflammatory pathway independently of Th2/IgE. In mycosis fungoides, IDECs are found in the epidermis, alongside elevated levels of malignant T lymphocytes.

#### 5.2.5. Inflammatory Dermal DCs (infDCs)

InfDCs accumulate in the skin in response to inflammatory injury in both human and murine models [5,53,111,112,113]. Human infDCs differentiate from classical circulating CD14^+^CD16^−^ monocytes*, which is why they are also referred to as monocyte-derived DCs (moDCs) [53,114,115,116]. In skin affected by psoriasis, Th1 and Th17 cells are found in close association with monocytes. This results in the differentiation of monocytes into DCs via GM-CSF and TNFα. These DCs then promote Th1 and Th17 immune responses, respectively [114,116]. Similarly, in atopic dermatitis, monocytes under Th2 influence differentiate into DCs capable of eliciting Th2-type immune responses [114]. MicroRNA-148a (miRNA-148a) regulates the differentiation of moDCs. Meng et al. demonstrated that increased expression of miRNA-148a in monocytes is present in patients with psoriasis, suggesting that it may serve as a therapeutic target [117]. MoDCs are involved in synthesising and secreting pro-inflammatory molecules, such as IL1, IL12, IL23 and TNFα, and stimulate CD4^+^ and CD8^+^ T cells [24,53].

Depending on their surface molecule profile and the role they perform, distinct subpopulations of infDCs have been identified: Tip-DCs, Slan-DCs and IFN-DCs.

Tip-DCs express and produce iNOS and TNFα. Tip-DCs have been demonstrated in vivo in human psoriatic skin lesions and in vitro in cultures of human moDCs [118,119]. They also play a key role in the development of chronic wounds, particularly diabetic foot ulcers and non-healing venous leg ulcers. In these conditions, they often remain ‘stuck’ in an activated state [120]. Tip-DCs alter the function of neutrophils and fibroblasts, and secrete high levels of proinflammatory cytokines that prevent tissue regeneration.

The Slan DC subpopulation (6-Sulfo LacNAc DC) has only been described in humans and is CD16^+^*, which places it on the monocyte line rather than the DC line [121]. The 6-sulfo-LacNAc glycan marker is specific to human Slan cells and non-classical human monocytes. This population grows preferentially in psoriasis or cutaneous lupus lesions, although small numbers can also be found in healthy skin [76,122,123,124,125]. Slan cell maturation occurs in an inflammatory context, followed by the synthesis of increased amounts of TNFα, IL1β, IL6, IL12 and IL23 via TLR7 and TLR8 ligands. The process by which Slan-DCs synthesise IL-23 is controlled by TNFα and IL-1β, which are also synthesised by Slan-DCs themselves (autocrine stimulation) [123,124,125,126].

The IFN-DC subpopulation has been characterised as a human subset generated in vitro from monocytes in the presence of IFN [115,116]. IFN-DCs express TLRs 7 and 8, enabling them to detect ssRNA, thereby intensifying Th1-type cytokine synthesis. Furthermore, exposure to ssRNA induces maturation of these cells [115,116,127]. Mature IFN-DCs produce IFNγ and IFNα, as well as IL-1β, IL-6, TNFα, IL-12, IL-23 and IL-27. The synthesis of IL-12 and IL-23 activates Th1 and Th17 cell subsets. These subsequently synthesise mediators that activate keratinocytes and macrophages, respectively, thereby activating the immune axes mediated by them [115,116]. In the presence of ssRNA, IFN-DCs demonstrate an enhanced capacity to stimulate the differentiation and activation of naïve T cells [115,127].

#### 5.2.6. Plasmacytoid DCs (pDCs)

pDCs are cells that have been described in both human and murine models. They have a morphology similar to that of plasma cells and are the primary producers of type I IFN, accounting for over 95% of production [10,128,129,130]. They are physiologically found in an immature state in the blood, and are also present in primary and secondary lymphoid organs. They are a specialised cell type of the innate immune response [128,130]. For a long time, their presence in the skin was considered pathological, initially being documented in conditions with dense lymphocytic infiltrates (e.g., cutaneous pseudolymphomas). They were later documented at higher frequencies in inflammatory dermatoses, such as psoriasis [128,131,132,133]. Despite being primarily associated with inflammatory conditions, recent studies have revealed the presence of pDCs in small numbers and in an irregular distribution pattern in healthy skin [10,82,134]. pDCs have precursors derived from bone marrow with dual myeloid and lymphoid origins [130,135]. Cutaneous pDCs differ from cDCs in terms of their role in the body, their embryological origin, and their lack of expression of the myeloid markers CD11c and CD1a. They express the following: BDCA2 (CD303)*, BDCA4 (CD304)*, CD123*, CD45RA* and HLA-DR*. pDC recruitment to the skin (in inflammation) involves CD62L, chemokine receptors, and ChemR23 (CMKLR1, chemerin receptor) [128,136,137,138], while pDC activation and maturation depend on TLRs [128,130,136]. pDCs play a pivotal role in innate immunity due to their intracellular expression of TLRs 7 and 9, and are involved in antiviral defence and immune tolerance [40,45,48,128,129,130]. In autoimmune settings, immune-tolerance is broken, as antimicrobial peptides (such as LL-37) can bind to and protect self-DNA. This forms immune complexes that facilitate uptake into endosomes. This enables aberrant TLR9-dependent type I IFN production by pDCs, which in turn drives autoimmune skin inflammation. This has been observed in conditions such as lupus erythematosus and psoriasis [40,45,47,48,128,131,133,139] (see Figure 3). pDCs activate cDCs via IFNα [131,136]. pDCs play a dual role in the immune system, as they can also induce immune tolerance in peripheral tissues by modulating the activity of Treg cells [140].

## 6. Other Cutaneous APC Populations

Langerhans-like cells***, ASDCs (Axl^+^ Siglec6^+^ dendritic cells)* and mature regulatory dendritic cells (mRegDCs)* are emerging DC populations with distinct features that reflect the expanding complexity of the skin dendritic compartment [12,15,16]. Their key characteristics, as revealed by single-cell studies, are summarized in Table 3.

## 7. Challenges and Emerging Concepts in Cutaneous APCs Research in 2025

The field of cutaneous APCs is constantly expanding, with recent research opening up several new avenues of investigation. New single-cell RNA sequencing techniques have revealed greater diversity within skin APC populations than was previously thought. Local factors in the skin microenvironment can also induce phenotypic and functional changes in these cells.

A key area of interest is the interaction between cutaneous APC subtypes, the skin and gut microbiome, and major inflammatory dermatoses, such as psoriasis, atopic dermatitis, hidradenitis suppurativa, acne vulgaris, and seborrheic dermatitis. Imbalances in the skin and gut microbiome can reshape DC subpopulations and the inflammatory networks involved in chronic dermatoses. Microbiome-modulating therapies, such as probiotics, postbiotics and microbiota transplantation, are emerging as strategies for preventing exacerbations and reducing comorbidities [141,142,143,144]. In atopic dermatitis, disruption to the epidermal barrier, microbial dysbiosis, and epithelial-derived cytokines such as thymic stromal lymphopoietin work together to drive the functional reprogramming of LCs and DCs towards a pro-allergic phenotype. This favours Th2-dominated immune responses, while impairing tolerogenic DC functions and sustaining chronic skin inflammation [10,73,110,144].

The mechanisms by which cutaneous APCs act in skin cancer and the tumour microenvironment are not yet clear. Given the frequency of carcinomas and the prognostic importance of melanoma, studies indicate that reprogramming cutaneous APCs to enhance their ability to stimulate effective immunotherapy is a promising approach. Recent data indicates that DC subsets in the tumour microenvironment can predict responses to immunotherapy and that signals derived from melanoma can suppress the activation of pDCs and cDC1s. This lends weight to the notion that reprogramming or targeting these cells could enhance the effectiveness of anti-tumour therapies [25,145,146,147,148,149].

Another important area of research could focus on the interaction between cutaneous APCs and dermatotropic viruses, as well as commensal and pathogenic skin bacteria. Recent research shows that viruses such as HSV and VZV, as well as commensal bacteria, modulate distinct subsets of skin APCs (LCs, cDC2s and Epi-cDC2s), thereby influencing local tolerance or immunity reactivation. Targeting these cells through skin vaccines or local immunotherapies is therefore emerging as a promising strategy for strengthening anti-infectious responses and preventing recurrences [6,100,150,151,152]. Furthermore, understanding the molecular and immune escape mechanisms of viral infections such as herpes simplex virus further emphasises the potential of DC-based interventions to overcome immune evasion and enhance long-term control [153].

A precise understanding of how cutaneous APCs participate in the activation of autoreactive T cells could form the basis for identifying potential therapeutic targets in cutaneous autoimmune diseases such as vitiligo, cutaneous lupus erythematosus, pemphigus vulgaris and psoriasis. The efficacy of targeting DCs and LCs with immunomodulatory molecules has recently been demonstrated in conditions such as cutaneous lupus erythematosus (e.g., litifilimab) and psoriasis (e.g., topical compounds and PD-L1 regulatory mechanisms). In lupus erythematosus, the abnormal sensing of self-nucleic acids by pDCs and myeloid DCs via endosomal TLRs leads to the sustained production of type I interferons. This promotes the activation of inflammatory DCs subsets and amplifies pathogenic Th1 and Th17 immune responses in both cutaneous and systemic disease. This provides the rationale for therapeutic strategies that target pDC activation and IFN-I signalling and confirm the pivotal role of APCs in activating autoreactive T cells [125,128,154,155,156,157,158,159,160].

Although it is clear that APCs are involved in neuroimmune responses in the skin, further research is required to determine their precise role in the perception of pain or itchiness in dermatological conditions. Cutaneous APCs can directly modulate nociceptive and pruritogenic activity by interacting with sensory fibres and inducing pruritus-specific receptors. This confirms their central role in neuroimmune circuits involved in pain and itching [102,161,162,163,164].

A key chapter could address the potential application of cutaneous APCs in cell and regenerative therapies, which play a vital role in treating burns and chronic ulcers, as well as in developing immunomodulatory skin grafts. Recently, genetic editing of DCs—for instance, through Ndrg2 knockout—has been associated with increased regenerative potential and accelerated healing of skin lesions. This opens up the possibility of using these cells in regenerative therapies and immunomodulatory skin grafts [120,165,166].

Lastly, a significant emerging challenge is harnessing cutaneous APCs to induce immune tolerance rather than classical immunity, particularly in allergic and autoimmune skin disorders. Epicutaneous and microneedle-based allergen delivery have recently shown promise in promoting tolerogenic DC phenotypes. Furthermore, targeting antigens through specific receptors, such as Langerin or DEC-205, on skin APCs, may provide novel vaccine strategies that achieve long-term immune regulation instead of pathogenic inflammation [167,168,169].

## 8. Discussion

This paper provides a comprehensive overview of the morphology and physiology of skin APCs in normal and pathological skin. It presents information on their origin, maturation and migration, as well as their key roles in innate and adaptive immunity. Thanks to modern immunohistochemical technologies, the different skin APC subtypes are becoming increasingly well characterised, and this paper describes them in terms of their interactions with the inflammatory microenvironment of the skin and the peripheral nervous system. This sheds new light on the role of the skin as an immune organ.

A thorough examination of the diversity of skin APCs was conducted. The APC subpopulations present in the skin are detailed, including the classical subsets of the human integument (LCs and cDCs), as well as those present in an inflammatory context (the aforementioned subsets, plus pDCs, IDECs and infDCs), while also incorporating recently described or refined populations such as DC3, Langerhans-like cells, ASDCs and mregDCs [5,10,11,12,15,16].

This perspective has been extended to include other skin APC populations as distinct subsets, as well as phenotypic and transcriptional variations within the dermal myeloid compartment. This paper differentiates the distinct DC3 subset from the broader category of CD14^+^ dermal DCs. The latter represents a heterogeneous population comprising both true DC3s and monocyte-derived DCs that predominantly appear in inflammatory contexts [12,13,14]. New populations with unique characteristics have also been identified, including Langerhans-like cells, ASDCs and mregDCs. These populations are involved in mediating inflammatory processes in the skin, reflecting the growing complexity of the skin dendritic compartment [12,15,16]. Additionally, the phenotypic variability of the dermal myeloid compartment is highlighted by the description of CD5^+^ and CD301b^+^ DCs, representing pro-inflammatory fractions within the cDC2 compartment [14,17,18].

The role of CD112 in the migration of skin DC populations has been highlighted, despite this being a rarely discussed topic in the current literature. A recent study demonstrated that CD112 facilitates the lymphatic migration of human dermal DCs under steady-state conditions, revealing an adhesion pathway that was previously unrecognised and that is relevant for cutaneous immune surveillance [32]. This paper provides detailed insights into the molecules involved in skin APCs migration. The recruitment and differentiation mechanisms of APC populations from myeloid and monocyte precursors under basal and inflammatory conditions are emphasised, with a particular focus on post-injury repopulation and dependence on cytokines such as GM-CSF and IL34. The significant connection between TLRs, pDCs, and the potential for triggering autoimmune diseases was emphasised, given that these cells are able to recognise self-nucleic acid fragments.

Additionally, the dual role of LCs in the skin microenvironment was discussed. It was highlighted that, depending on the signals they receive, LCs can exhibit either immunostimulatory or immunoregulatory properties [84]. Furthermore, the paper emphasised the collaborative relationship between APC populations, for instance, between LCs and dermal DCs [83,84]. The neuro-immune dialogue in the skin is complex and based on bidirectional interactions between the peripheral nervous system and skin APC. Neurotrophins secreted by LCs influence nerve fibre density. CGRP modulates the cDC2-dependent Th17 response and IL23 synthesis [102,104,105].

The paper explored the role of skin APC in the immunological mechanisms of psoriasis and other inflammatory skin diseases, with a particular focus on SlanDC population. SlanDCs originate from the monocyte lineage and are characterised by the expression of CD16 [121]. SlanDCs can secrete IL-23, TNF-α and IL-1β, all of which are essential for the Th17 inflammatory response [123,124,125,126]. Details were also presented on mregDCs (LAMP3^+^ BIRC3^+^ mature migratory DCs), which were recently identified in patients with psoriasis [12]. These cells play a key role in maintaining chronic inflammation by increasing the production of IL15 and IL32. Single-cell analyses have demonstrated the presence of mregDC LAMP3^+^ cells in inflamed skin, including in cases of psoriasis and atopic dermatitis, thus confirming their local immunoregulatory role [12]. However, the literature is divided on whether these cells should be interpreted as a stable DC subset with its own transcriptomic profile, or as a maturation and migration programme that other DC subsets can temporarily adopt under the influence of the microenvironment [170]. Considering the aforementioned points, psoriasis involves subsets of APCs that trigger the disease, such as pDCs, as well as subsets that amplify the disease, such as moDCs and Tip-DCs. In atopic dermatitis, the subsets that trigger the disease are LCs and cDC2s, while the subsets that amplify it are IDECs [10,92,118,133].

Inflamed skin exhibits changes in function and cell phenotype (plasticity), as well as the presence of numerous APC subtypes. We would argue that this redundancy is most evident in expressing overlapping PRRs for antigen detection and processing, but is reduced when highly specialised antigen presentation occurs (like LCs T17 responses against Candida albicans) and the inflammation polarization phase [171]. Disease can be altered by selective depletion, such as c-Jun/AP-1 deletion in DCs, which reduces imiquimod-induced skin inflammation [49].

The potential clinical and therapeutic implications of skin APCs were also discussed. Novoszel et al.’s paper uses mouse models (imiquimod-induced and genetic), human psoriatic skin, and human DCs ex vivo to demonstrate that c-Jun in DCs drives CCL2 (pDC recruitment) and IL-23 (Th17 axis). Blocking JNK/c-Jun improves psoriasiform disease in mice and reduces these cytokines in TLR 7/8-stimulated human DCs [49]. This mechanism provides evidence that AP-1 proteins are involved in the pathogenesis of psoriasis. However, AP-1/c-Jun targeting remains at the preclinical stage in dermatology. To date, there have been no clinical trials specifically targeting c-Jun/AP-1 in dendritic cells (DCs) for psoriasis. The role of IL34 and CSF-1R in maintaining DC homeostasis was discussed, as was the possibility of manipulating these pathways in future therapies. Additionally, microRNA-148α was presented as a potential biomarker and therapeutic target in psoriasis due to its influence on the differentiation of inflammatory DCs. Although the feasibility of selectively targeting APCs could be debated, the overlap of markers between subsets and the compensation process makes this approach challenging.

## 9. Conclusions

Due to their complex role in immune defence and their ability to regulate lymphocyte- mediated immune responses, alterations in skin APC, such as changes in migratory capacity, degree of maturation, or ability to present antigens, can lead to the dysregulation of physiological immune functions and the onset of chronic immune and inflammatory diseases. This article is the first skin-related approach that extensively discusses the morphofunctional diversity of skin APCs, their interactions with the skin microenvironment and their migration and activation mechanisms. Notably, it reveals new correlations with inflammatory skin diseases. The lines of research it opens up are likely to have an impact in the near future by providing potential therapeutic targets for major dermatological conditions.

## Figures and Tables

**Table 1:** Chemokine–Receptor pairs involved in mature DCs migration from tissue to lymph nodes.

Chemokine Receptor	Chemokine
CCR7	CCL19
CCR7	CCL21
CXCR4	CXCL12
CCR2	CCL2
CCR6	CCL20

**Table 2:** Key characteristics of DC3 cells (Notes: * human).

Subset	Where?	Key Markers	Key Cytokines	Signature Functions	Pathology Highlights
DC3 (CD1c^+^ CD14^+^ type 3 dendritic cells) * [12,14,53,79,90,106]	Circulation Dermis (steady-state and inflamed)	CD1c^+^, CD14^+^, CD11c^+^, CD163^+^, HLA-DR^+^ CD88^−^, CD5^−^ TLR 2/4/5/6/8/10	IL1β, IL23, TNFα, IL6, IL8, IL10, IL12, IL20, GM-CSF, MCP, TGFβ	Recognize and present antigens Activates T cells (Th1, Th2, Th17). Mediates the interaction between T cells and B cells/plasma cells. Activates humoral immune response. Induces Treg and Tfh cells (immunoregulatory properties). Shows marked expression of genes involved in IL17-dependent signalling and neutrophil recruitment, such as IL23A and CXCL2.	Inflammatory cells found in increased numbers in the blood of SLE patients and in the lesion skin of psoriasis, where they secrete IL1β and IL23A, and promote Th17 axis. Their proportion is higher in psoriasis in the lesion skin compared to the non-lesion skin. In SLE patients they are correlated with disease activity

**Table 3:** Key characteristics of new emergent DC populations (notes: * human, *** murine).

Subset	Where?	Key Markers	Key Cytokines	Signature Functions	Pathology Highlights
Langerhans-like cells *** [15]	Circulation, Cutaneous lymph nodes Dermis (steady-state)	CD207^+^, CD326^+^		High migratory capacity towards the lymph nodes Induce cutaneous tolerance to haptens	Tolerance induction in contact hypersensitivity and inflammatory skin diseases should be attributed to this dermal LC-like subset Potential therapeutic interest: targeting the LC-like migratory DCs that regulate tolerance
ASDCs (Axl^+^ Siglec6^+^ dendritic cells) * [16]	Circulation Peripheral tissues, including skin (inflamed)	Axl^+^, Siglec6^+^		Pro-inflammatory phenotype T cell activation Initiation of acute immune responses	In the human cutaneous inflammation model, ASDCs are among the first DCs recruited to the inflamed site
Mature regulatory DCs (mRegDCs) * [12]	Dermis (steady-state and inflamed)	LAMP3^+^, BIRC3^+^	IL15, IL32	Mature, migratory population Immunomodulator DC differentiation Lymphocyte migration (secondary to intense expression of IRF4 and CCL17 genes)	Prominent in psoriasis and atopic dermatitis

## Data Availability

No new data were created or analyzed in this study. Data sharing is not applicable to this article.

## Abbreviations

The following abbreviations are used in this manuscript:

DCs	Dendritic cells
APCs	Antigen-presenting cells
MHC	Major Histocompatibility Complex
LCs	Langerhans cells
CD	Cluster of differentiation
DC3	Type 3 DCs
ASDCs	Axl+ Siglec-6+ dendritic cells
mregDCs	Mature dendritic cells enriched in immunoregulatory molecules
TNFα	Tumor necrosis factor alpha
IL	Interleukin
CCL	C-C motif chemokine ligand
CCR	C-C chemokine receptor
CXCL	C-X-C motif chemokine ligand
CXCR	C-X-C chemokine receptor
Th	T helper
TCR	T-cell receptor
LFA	Lymphocyte function-associated antigen
ICAM	Intercellular adhesion molecule
PRRs	Pattern recognition receptors
TLRs	Toll-like receptors
CLRs	C-type lectin receptors
HIV	Human immunodeficiency virus
CLEC7A	C-type lectin domain containing 7A
DC-SIGN	Dendritic cell-specific ICAM-3-grabbing non-integrin
Treg	Regulatory T cell
PAMPs	Pathogen-associated molecular patterns
DAMPs	Damage-associated molecular patterns
NF-kB	Nuclear factor kappa-light-chain-enhancer of activated B cells
IRF7	Interferon regulatory factor 7
MyD88	Myeloid differentiation primary response 88
IFN	Interferon
AP-1	Activator protein-1
TRAF-6	TNF receptor-associated factor 6
TAK-1	TGF-beta-activated kinase 1
JNK	c-Jun N-terminal kinase
TGFβ	Transforming growth factor beta
CSF-1	Colony-stimulating factor 1
BMP-7	Bone morphogenic protein 7
AXL	AXL receptor tyrosine kinase
cDCs	Conventional DCs
CDPs	Myeloid common progenitors of DCs
FLT3	Fms-like Tyrosine kinase 3
FLT3L	Fms-like tyrosine kinase 3 ligand
GM-CSF	Granulocyte-macrophage colony-stimulating factor
CSF2	Colony-stimulating factor 2
CSF2R	Colony-stimulating factor 2 receptor
cDC1s	Conventional type 1 dermal dendritic cells
cDC2s	Conventional type 2 dermal dendritic cells
BDCA3	Blood dendritic cell antigen 3
CLEC9A	C-type lectin domain family 9 member A
Cadm1	Cell adhesion molecule 1
XCR1	X-C motif chemokine receptor 1
BDCA	Blood dendritic cell antigen
CSF-1R	Colony-stimulating factor 1 receptor
CSF1	Colony-stimulating factor 1
MIP3α	Macrophage inflammatory protein-3 alpha
MMP	Matrix metalloproteinase
L1CAM	L1 cell adhesion molecules
Tfh	T follicular helper
RANK	Receptor activator of nuclear factor kB
RANKL	Receptor activator of nuclear factor kB ligand
NGF	Nerve growth factor
GDNF	Glial cell line-derived neurotrophic factor
PGE2	Prostaglandin E2
CGRP	Calcitonin gene-related peptide
IDECs	Inflammatory epidermal dendritic cells
InfDCs	Inflammatory dendritic cells
moDCs	Monocyte-derived dendritic cells
TipDCs	TNFα and iNOS-producing dendritic cells
iNOS	Inducible nitric oxide synthase
Slan-DC	6-Sulfo LacNAc dendritic cells
IFN-DC	Interferon-induced dendritic cell
ssRNA	Single-stranded RNA
pDCs	Plasmacytoid dendritic cells
HLA-DR	Human leukocyte antigen-DR isotype
ChemR23	Chemokine-like receptor 1 (CMKLR1)
DNA	Deoxyribonucleic acid
RNA	Ribonucleic acid
MCP	Monocyte Chemoattractant protein
SLE	Systemic lupus erythematosus
LAMP3	Lysosome-associated membrane glycoprotein 3
BIRC3	Baculoviral inhibitor of apoptosis repeat-containing protein 3
HSV	Herpes Simplex Virus
VZV	Varicella-Zoster Virus
Epi-cDC2s	Epidermal conventional type 2 dermal dendritic cells
PD-L1	Programmed death-ligand 1
DEC-205	CD205
